# Medium cut-off dialyzer for middle molecular uremic toxins in AKI and chronic dialysis

**DOI:** 10.1007/s40620-023-01771-0

**Published:** 2023-10-16

**Authors:** Marco Fiorentino, Francesco La Fergola, Silvia De Rosa

**Affiliations:** 1 Nephrology, Dialysis and Transplantation Unit, Department of Precision and Regenerative Medicine and Ionian Area (DiMePRe-J), University of Bari “Aldo Moro”, Bari, Italy; 2 Centre for Medical Sciences - CISMed, University of Trento, Via S. Maria Maddalena 1, 38122 Trento, Italy; 3 Anesthesia and Intensive Care, Santa Chiara Regional Hospital, APSS Trento, Trento, Italy

**Keywords:** Continuous veno-venous hemodialysis, Cytokine clearance, EMiC2 filter, Middle cut-off, Removal, Uremic toxins

## Abstract

Uremic toxins accumulate in patients affected by renal failure and can deposit in different organs, including the kidneys and heart. Given their physicochemical characteristics, uremic toxins can contribute to organ dysfunction due to several pathobiological actions at cellular and molecular levels. Several uremic compounds have been described in serum and plasma from patients with acute kidney injury (AKI) and kidney failure; they are usually classified based on their molecular size and protein-binding properties. In this scenario, new dialytic approaches have been proposed in the last few years with the aim of improving uremic toxin removal. Recent studies which focused on the use of medium cut-off membranes in patients on chronic hemodialysis have shown a discrete ability to remove β2-microglobulin and other middle molecules, such as kappa and lambda free light chains, complement factor D and α1-microglobulin. However, current evidence is mainly based on the impact on short-term outcomes and, consequently, longer observational studies are necessary to confirm the efficacy and safety of the medium cut-off dialyzer. Here we present the state-of-the-art on the clinical application of medium cut-off membranes in AKI and chronic dialysis patients.

**Graphical abstract:**

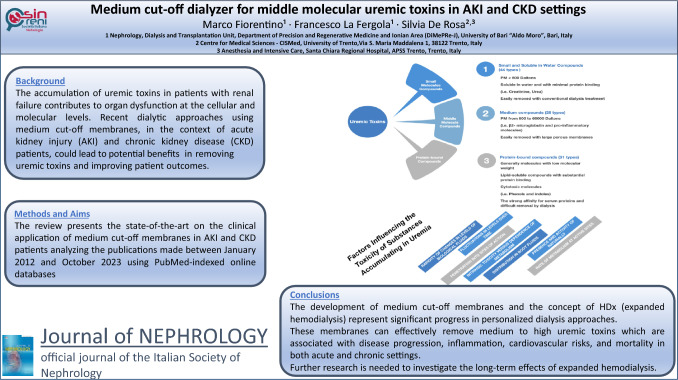

## Introduction

Recent advances in the pathophysiology of kidney disease and the advent of new treatments to control modifiable risk factors have limited both progression to end stage kidney disease (ESKD) and cardiovascular risk in patients with chronic kidney disease (CKD). In addition, gut microbiota dysregulation has recently been described as one of the main factors associated with CKD progression, contributing to worsening of renal function and fluid, electrolyte, hormonal, and metabolic abnormalities [1]. In the context of kidney failure and uremia, several solutes are retained, exhibiting different physicochemical characteristics and specific biologic effects [2]. Uremic toxins are typically generated from protein metabolism and may accumulate in the bloodstream in patients with renal dysfunction, reaching different organs (including the kidneys and heart), affecting their biologic functions. Uremic syndrome is therefore characterized by the accumulation of several noxious substances, playing a prominent role in inducing endothelial dysfunction [3]. The recent introduction of novel dialytic membranes has led to a paradigm change in the management of uremic syndrome [4]. Recent studies on the use of the medium cut-off membranes in chronic hemodialysis (HD) patients have shown the ability to remove β2-microglobulin and larger middle molecules, such as kappa and lambda free light chains, complement factor D, and α1-microglobulin [5]. Expanded hemodialysis with medium cut-off membranes was designed to improve the permeability of the dialyzers, the removal of larger molecules and, consequently, clinical outcomes [6]. In addition, the use of such membranes could be critical in specific settings, such as sepsis or rhabdomyolysis, that are associated with the development of acute kidney injury (AKI). To date, scientific evidence supporting the use of medium cut-off membranes is mainly based on retrospective and prospective studies with limited sample size and short-term outcomes; in this setting, longer observational studies are essential to confirm the efficacy and safety of the medium cut-off dialyzer. Herein, we discuss the state-of-the-art on the clinical application of medium cut-off membranes, describing their effects on removing uremic toxins, alleviating inflammation and improving quality of life and cardiovascular risk in AKI and CKD patients.

## Methods

As a basis of this narrative review, we searched for publications between January 2012 and October 2023 indexed on PubMed. We selected relevant publications involving adults (defined as older than 18 years) and written in English. We used the terms “(uremic toxins) AND (MCO membrane) OR (Expanded hemodialysis) OR (High-Flux) OR (Super High-Flux) OR (Online Hemodiafiltration)”.

We used a combination of the terms and cross-referenced the publications to remove duplicates. We analyzed the references in each selected article to ensure that other publications not retrieved by the initial search were not missed. Since this was not a systematic review, we did not record the total number of publications analyzed, multiple publications and publications written in languages other than English.

### Uremic toxins in AKI and CKD

**Fig. 1:**
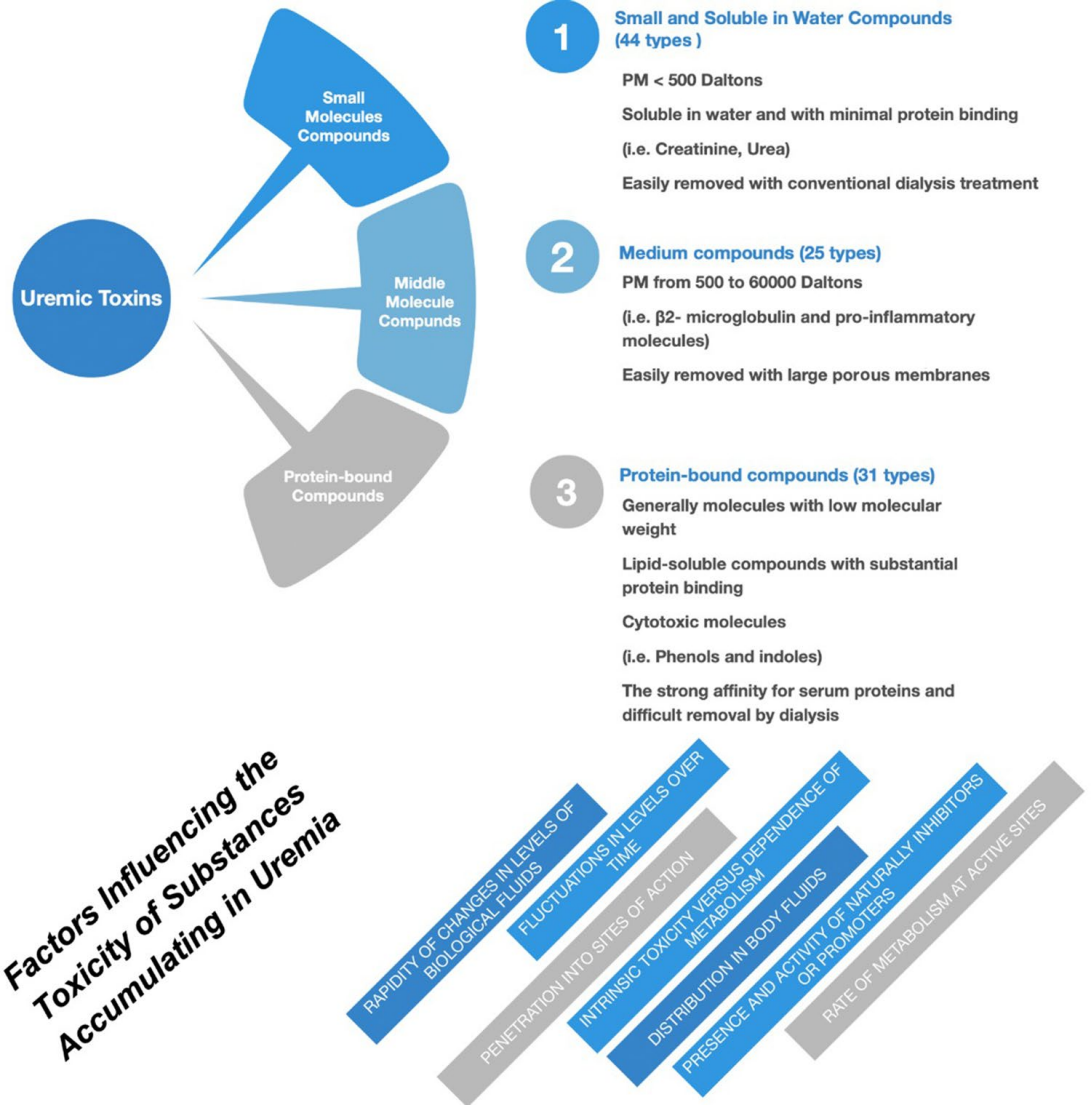
Classification of uremic toxins

According to the European Uremic Toxins (EUTox) Work Group [7], uremic toxins are defined as harmful compounds that accumulate in the body during periods of renal function decline [8, 9]. Uremic toxins progressively increase in CKD, promoting several biochemical and functional changes [10]. Uremic toxins may be classified according to their chemical nature (inorganic/organic), molecular mass/volume (small/middle/large), their distribution in body fluids (hydrophilic/lipophilic/bound to plasma proteins), pattern of removal by dialysis, biological properties and potential to produce clinical symptoms. The EUTox database currently lists more than 153 uremic toxins, with numbers increasing over time [9]. Based on chemical and physical characteristics, uremic toxins are conventionally divided into 3 groups, essentially on the basis of their removal by dialysis (Fig. 1):

1. Small water-soluble compounds (< 500 Da), with minimal protein binding. These molecules, including urea and creatinine, are easily removed by hemodialysis. Of the 90 molecules evaluated by EUTox, 68 belong to this group.
2. Medium compounds (molecular weight > 500 Da). β2–microglobulin (β2M) and leptin are the prototypes of this group; these toxins are partially removed by dialysis membranes with high permeability (large pores).
3. Protein-bound compounds (including phenols and indoles). These compounds originate from the metabolism of dietary amino acids. In patients on chronic hemodialysis the removal is limited to the unbound fraction and occurs by convection, but is independent of dialyzer pore size. The EUTox classification does not describe the toxicity of these compounds.

Table 1 shows the main characteristics of some uremic toxins. The Massry/Koch requirements for the identification of an ‘‘authentic’’ uremic toxin are based on chemical identification and characterization, levels and quantitative analysis in biological fluids, relationship with one or more uremia symptoms and reproducibility of uremic manifestations after administration of the toxin in animals or healthy subjects [11]. Figure 1 shows factors potentially influencing the toxicity of substances accumulating in uremia.

**Table 1:** Main uremic toxin characteristics

Molecule	Molecular weight	Protein binding	Origin	Mechanism	Dialyzability
Low molecular weight compounds not bound to protein					
Creatine	131 Da	Free water soluble	Endogenous metabolites of furan fatty acids	Direct interaction with free oxygen radicals, which can induce cell damage	Mainly extracted from plasma during HD
Creatinine	113 Da	Free water soluble	Waste product generated by muscle metabolism	Chloride channel blocking and reduction of the contractility of cultured myocardial cells	Mainly extracted from plasma during HD
Guanidine	59 Da	Free water soluble	Protein breakdown product	Inhibition of neutrophil superoxide production, suppression of natural killer cell response to interleukin-2, abnormalities of vascular repair, increased manifestations of atherosclerosis, including leukocyte activation	Mainly extracted from plasma during HD
Oxalate	90 Da	Free water soluble	Toxin synthesized during purine metabolism	Enhancement of atherogenesis; Immunomodulation through induced synthesis of cytokines, chemoattractants, and other inflammatory signal molecules causing degradation of IκBα in proximal tubular cells; unfavorable impact on mitochondrial function	Efficient modern HD is usually enough to prevent intratissue deposition
Urea	60 Da	Free water soluble	Waste generated from the breakdown of unused proteins in the liver	Molecular changes related to insulin resistance, generation of reactive oxygen species, apoptosis, and disruption of the intestinal protective barrier	Mainly extracted from plasma during HD
Uric acid	168 Da	Free water soluble	The end product of purine metabolism	Contribution to the genesis and progression of cardiovascular disease and chronic kidney disease	Removal in HD (during 1 HD session on average 1 g uric acid is eliminated)
Middle molecule compounds					
Adiponectin	30,000 Da	N/A	Protein hormone mainly produced by adipocytes	Anti-inflammatory (suppression of M2 to M1, macrophage differentiation and inhibition of the production of proinflammatory cytokines), anti-dyslipidemic and anti-diabetic effects; Changes in arterial stiffness	It is not removed readily by conventional HD, but it might be removed by high flux and HDF. Due to undialyzable characteristics and decreased excretion from impaired renal function, adiponectin level is elevated in patients receiving HD and PD
Complement factor D	26,750 Da	N/A	Serine protease synthesized by adipocytes	Enhancement of alternative pathway activation	Some dialysis membranes (for example, AN69) adsorb complement factor D
Cystatin C	13,300 Da	N/A	A non-glycosylated protein produced continuously by all cells in organs/tissues	It is filtered in the renal glomeruli and completely reabsorbed by the renal tubules. Alterations of serum cystatin C were considered an early renal marker in diabetic patients, cardiovascular diseases kidney transplantation, hyperthyroidism, cancer	High-flux membranes remove up to 50% of cystatin C
Interleukin-1β	32,000 Da	N/A	Produced as an inactive 31 kDa precursor, termed pro-IL-1β, in response to molecular motifs carried by PAMPs	Increased in diseased coronary arteries and correlates with plaque severity. IL-1 beta has also been associated with left ventricular hypertrophy in dialysis patients	High cut-off hemofiltration techniques are more efficient in removing cytokines than standard techniques
Tumor necrosis factor alpha (TNFα)	26,000 Da	N/A	Pro-inflammatory cytokine synthesized in large amounts by activated macrophages and T cells, but also by stressed epithelial and other cells	It stimulates the release of inflammatory cytokines, upregulates the expression of endothelial adhesion molecules and chemokines, promotes cell death and decreases the expression of the anti-inflammatory and anti-aging protein Klotho	High cut-off hemofiltration techniques are more efficient in removing cytokines than standard techniques
β2- microglobulin	11,818 Da	N/A	Non-glycosylated polypeptide that forms the non-variable light chain of the Class 1 major histocompatibility complex	Retention of β2M produces a disease related to the deposition of β2M amyloid fibrils around large joints such as shoulders and hips	Poorly removed by HD Large-pore membranes adsorb substantial amounts of β2- microglobulin
Low molecular weight compounds bound to protein					
3-Carboxy-4-methyl-5-propyl-2-furanpropionate (CMPF)	240 Da	More than 95% to albumin	Metabolite of furan fatty acid and a marker of fish oil intake	Interaction with free oxygen radicals, which can induce cell damage	Clearance of the unbound fraction may be increased by the use of HDF, especially post-dilutional modes. “Leaky” dialysis membranes increase the clearance of such substances bound to plasma proteins
Hippuric acid	179 Da	34–40% bound to albumin	Gut-derived, protein-bound UT	Hippuric acid may enhance toxicity of protein-bound drugs and uremic solutes by competing for protein binding	Clearance of hippuric acid by HD is 64%
Homocysteine (Hcy)	135 Da	Covalently bound to albumin	Sulfur-containing amino acid, produced by the demethylation of dietary methionine	Hcy increases the proliferation of vascular smooth muscle cells (atherosclerosis), endothelial dysfunction, generation of reactive oxygen species and enhancement of thrombogenicity	Partly removed during HD. Agents such as N-acetyl cysteine and mesna may displace Hcy from the protein binding site, facilitating removal during HD
Indole-3-acetic acid	175 Da	80% bound to albumin	Uremic indolic toxin derived from the metabolization of dietary tryptophan by the gut microbiota	Agonist of the transcription factor AhR which regulates vascular inflammation, oxidative stress, and atherosclerosis	Partly removed during HD, with a reduction rate of about 45% during the dialysis session. Removal of the protein-bound compounds during HD is limited and not enhanced during treatment with high-flux membranes
Indoxyl sulfate	212 Da	95% bound to albumin	A part of the dietary protein-derived tryptophan is metabolized into indole that is metabolized to indoxyl sulfate in the liver	It stimulates progressive tubulointerstitial fibrosis and glomerular sclerosis by increasing the expression of transforming growth factor-beta1, a tissue inhibitor of metalloproteinase-1 and proalpha 1 (I) collagen, leading to a further loss of nephrons	32% cleared through dialysis
p-Cresyl sulfate (PCS)	187 Da	90% bound to albumin	Plasma PCS concentrations are determined mainly by intestinal uptake of p‐cresol, metabolism of p‐cresol to PCS, and renal excretion of PCS	Accumulated PCS plays a role in activating leukocyte radical production, disturbing the renin–angiotensin–aldosterone system, promoting renal tubular cell damage, and interfering with insulin signaling pathways	64% cleared thought dialysis

*β2M* β2-microglobulin, *Da* Dalton, *HD* hemodialysis, *HDF* hemodiafiltration, *PCS* p-cresyl sulfate, *UT* uremic toxin

Although uremic toxins are usually filtered and excreted by the kidneys, they may accumulate in the setting of CKD and exert their uremic effects on various systems [12]. Their increased concentration is involved in the onset of several renal and non-renal complications that usually affect patients with kidney failure [13]. The accumulation of middle molecules are thought to participate in immune dysfunction, metabolic dysfunction (anorexia), inflammation and cardiovascular injury (cardiac hypertrophy, atherogenesis) [13, 14]. In addition, some of these compounds may accumulate in the central nervous system, and are involved in neurocognitive impairment  [15].

While the role of protein-bound uremic toxins in CKD is well established, limited information is available in the AKI setting [16]. Acute kidney injury is a serious and frequent condition predominately affecting critically ill patients and characterized by a sudden decrease in glomerular filtration rate and urine output due to tubular and endothelial injury [17]. The duration and severity of AKI episodes are major determinants of the risk of progression to CKD [18, 19]. Retention of some uremic toxins may contribute to organ dysfunction, impacting on mortality and renal recovery rate [16].

### Uremic toxin removal by extracorporeal treatments

The removal of protein-bound uremic toxins represents a challenge in patients with kidney falure, and several extracorporeal blood purification strategies have been proposed to improve toxin removal [20]. Conventional hemodialysis is the main extracorporeal depuration technique, effective in eliminating small water-soluble compounds, while the removal of middle molecules and protein-bound toxins is limited (reduction rate < 30–35%), due to the protein-bond and the pore cutoff of low-flux membranes, that prevent albumin loss [20, 21]. Moreover, the increase in the number of dialysis sessions and/or in the treatment time may improve small and middle molecule removal, but not removal of protein-bound molecules [22].

In this scenario, the introduction of high-flux membranes and the development of hemodiafiltration (HDF) led to increased clearance of middle molecular weight molecules compared to conventional hemodialysis by enlarging the pore size and increasing convection [20, 23]. High flux membranes are characterized by increased permeability, with larger pore size and increased ultrafiltration coefficient [24]. However, albumin and mico-nutrient loss should be taken into account; in addition, the ability to remove protein-bound uremic toxins still remains limited [25]. Similarly, Lesaffer and co-workers compared the impact of high and low flux membranes on protein-bound uremic toxin removal and did not observe any significant differences (34.4% vs. 36.3% for indoxyl sulfate, 32% vs. 28% for p-cresyl sulfate) [21].

High cut-off membranes showed interesting results in protein-bound toxin removal, since they have larger pores able to remove molecules with a molecular weight up to 60 kDa. High cut-off membranes have been considered in the treatment of patients with acute kidney injury secondary to multiple myeloma [26] and severe sepsis [27]. However, a significant amount of albumin loss limits their routine use in chronic patients [28].

More recently, the introduction of medium cut-off membranes allows a dialytic approach called expanded hemodialysis, characterized by an improved ability to remove middle and large molecules, thereby improving chronic inflammatory status and clinical outcomes [5, 6, 29].

### Medium cut-off membranes and the concept of Expanded Hemodialysis

Medium or high cut-off membranes have recently been introduced in the clinical practice [30, 31]. These membranes are usually composed of polyarylethersulfone and polyvinylpyrrolidone, reaching a surface area of at least 1.6 m^2^ (the most commonly employed are Theranova 400 and Theranova 500, with an effective surface area of 1.7 m^2^ and 2.0 m^2^, respectively). Furthermore, they present tighter pore distribution with larger pore size compared to high flux, low flux and high cut-off membranes [30]. The pore radius is about 5 nm, which becomes about 3.5 nm after contact with blood, enough to allow the removal of uremic toxins up to 45 kDa. Pore size and distribution provide a peculiar permeability profile to medium cut-off membranes, while the sieving coefficient curve is an empirically derived function that depends on these two features [32]. The sieving curve of medium cut-off membranes is steep, characterized by high molecular weight retention onset and high molecular weight cut-off values. The point in the curve where the sieving coefficient is 0.9 determines the molecular weight retention onset (the point at which only 10% of solute is retained) [30, 32, 33]. The point in the curve in which the sieving coefficient is 0.1 determines the molecular weight cut-off (the point at which 90% of solute is retained). The molecular weight retention onset and the molecular weight cut-off of medium cut-off membranes are very close to each other, with a cut-off value similar to that of high flux membranes; however, this value is lower than albumin weight, leading therefore to lower albumin loss [30, 33]. Conversely, the retention onset differs as compared to high flux membranes: the retention onset for medium cut-off membranes is close to 12 kDa, while for the high flux membrane it is close to 1200 Da. This especially steep curve leads to increased removal of uremic toxins in the middle/high molecular weight range, with marginal leakage of albumin [34]. Moreover, manufacturers succeeded in reducing the thickness and the inner diameter of the fibers (from the standard 200 µm to 180 µm), resulting in an improvement of membrane permeability and dialysis efficiency due to a larger number of fibers in a more compact dialyzer and increasing the wall shear rate and optimizing blood flow [35]. In addition, the improvement of solute transfer is also related to internal filtration and back filtration [36]. Internal filtration predominates in the proximal part of medium cut-off membranes and allows higher convective rates compared to standard membranes, leading to increased removal of middle molecules, typically characterized by low diffusion coefficients [36]. Conversely, back filtration mainly occurs in the distal part of the dialyzer, and it is useful for compensating for the excessive filtration obtained in the proximal part without need for reinfusion (as in HDF) [30, 36]. The ultrafiltration control system of the dialysis machine regulates the process and provides the exact amount of net filtration required for the scheduled weight loss [36]. The advantage is that this treatment does not require significant pre- or post-dilution substitution fluid and high performing vascular access in order to guarantee a higher blood flow. The peculiar properties and inner filtration-back filtration mechanism allow the medium cut-off membranes to enhance the removal of middle molecules compared to high flux membranes.

The term expanded hemodialysis has been introduced to define hemodialytic treatments performed with medium cut-off dialyzers because of their ability to increase the range of molecular weight of uremic toxins removed. Standard dialysis machines (with UF control system) can perform extended dialysis without specific software or replacement solutions and with standard parameters (blood flow ≥ 300 mL/min and a dialysate flow 500 mL/min) [33, 37]. In expanded hemodialysis, the convective clearance (K) results from the product of UF rate (Qf) and sieving (S) of a selected solute (K = Qf × S). With medium cut-off membranes, high sieving for medium and large molecules is the key for achieving high convective clearance with lower convective volume. In HDF, the high convective clearance is reached by increasing Qf thanks to the combined pre- and post-dilution configuration in HDF, however, online HDF is not approved in many countries. Conversely, expanded hemodialysis achieves a high level of clearance for molecules like β-2microglobulin and free light chains, (molecular weight of 22.5 and 45 kDa for kappa and lambda, respectively) [29]. On expanded hemodialysis, however, albumin loss is not negligible, and is estimated between 1.2 and 3.5 g per dialysis session with different medium cut-off membranes [38]. While in healthy individuals hepatic synthesis may compensate the losses, they may be relevant in elderly and frail patients. Furthermore, albumin leakage may allow removal of protein bound uremic toxins, like indoxyl sulfate and p-cresyl sulfate [39] that are not otherwise removed due to their binding to albumin, despite their low molecular weight (< 500 Da) [4]. In addition, the removal of inflammatory cytokines (IL-6, TNF-α) may be an added value of expanded hemodialysis [40, 41].

### Effects of expanded HD on uremic toxin removal compared to high-flux HD and HDF

Several studies focused on the efficiency and safety of medium cut-off membranes compared to conventional HD and/or HDF. A detailed list of the main clinical studies is reported in Table 2. Overall, these studies reported increased performance of such membranes compared to high flux dialyzers, although this higher reduction ratio has not clearly been associated with long-term clinical outcomes, possibly due to the small sample sizes. Recently, a systematic review and meta-analysis of 18 prospective interventional studies including a total of 853 patients with ESKD confirmed the safety and efficacy of medium cut-off membranes compared to high flux-HD (increased reduction ratio of β-2microglobulin, kappa and lambda free light chains), while these effects are not evident compared to HDF; no significant differences in albumin loss were reported as compared to HDF [42]. β-2microglobulin has a molecular weight of 11 kDa and represents the standard marker of middle molecule. Middle cut-off membranes increase the reduction rate of β-2microglobulin as compared to high flux-HD and HDF [5, 43–46]. However, rebound after expanded hemodialysis discontinuation was reported as with HDF [43], and long-term effects were not studied. Similarly, increased serum levels of kappa and lambda free light chains are associated with adverse outcomes in HD patients [47]. In a randomized controlled trial performed by Weiner et al., including 172 ESKD patients, the use of the Theranova 400 filter showed an increased reduction rate of both kappa and lambda free chains compared to standard high flux-HD [46]. Similar results were also described in 3 other randomized controlled trials [5, 45, 48]. In addition, conflicting results are available concerning removal of protein-bound toxins, including homocysteine, indoxyl sulfate and p-cresyl sulfate. Belmouaz et al. reported increased homocysteine removal with medium cut-off membranes compared to high flux-HD associated with moderate hypoalbuminemia [5]. However, the tRial Evaluating Mid cut-Off Value membrane clearance of Albumin and Light chains in HemoDialysis patients (REMOVAL-HD) did not show significant changes in indoxyl sulfate, sp-cresyl sulfate, fetuin-A, endogenous calciprotein particles (CPP-1 and CPP-2) in 89 ESKD patients [49]. New middle cut-off membranes have recently been developed, increasing the therapeutic options. The comparison between various medium cut-off membranes (Phylther 17-SD, Vie-18X, Elisio HX19 and Theranova 400) in a small prospective study carried out on 23 HD patients did not show significant differences in albumin loss. A new device (Elisio-17HX) with an inner diameter of 200 µm and a wall thickness of 40 µm, shows similar results to Theranova membranes. In a randomized controlled pilot study including 6 maintenance hemodialysis patients, Elisio-17HX was less efficient compared to Theranova though with a lower albumin loss [50].

**Table 2:** Main studies focusing on the effect of medium cut-off membranes on middle molecule removal

Study ID	Year	Study design	Pts	Main findings
Zickler et al. [41]	2017	Randomized crossover trial, MCO-HD vs. HF-HD	48	After 4 weeks, reduced expression of TNF- α and IL-6 mRNA with MCO treatments. Reduced plasma levels of several cytokines, kappa and lambda FLCs
Kirsch et al. [44]	2017	Prospective, open-label, controlled, randomized crossover pilot study; MCO-HD vs. HF-HD and HDF	39	Reduction ratios of λFLC were greater for MCO. Clearances of α1-microglobulin, complement factor D, κFLC and myoglobin were greater with MCO with moderate albumin loss
Reque et al. [78]	2019	Prospective study, cross-over design, online HDF vs. MCO-HD	8	Increased reduction rate of myoglobin and prolactin with HDx compared to HDF, no differences in reduction rate of urea and β2-microglobulin
Maduell et al. [79]	2019	Prospective, single-center study, MCO-HD vs. 8 different dialyzers in HDF	22	No significant differences in the RRs of small and middle molecular range molecules between MCO vs. OL-HDF
Garcia-Petro et al. [80]	2018	Cross-over study, analysis of 3 single mid-week dialysis sessions for 3 consecutive weeks; HF-HD vs. MCO-HD	18	Increased reduction in β2-microglobulin, myoglobin, prolactin, α-1 glycoprotein, with limited albumin loss
Kim et al. [81]	2019	Clinical trial, analysis of midweek dialysis for 3 consecutive weeks between HF-HD vs. HDF vs. MCO-HD	6	MCO-HD showed greater RRs for myoglobin and λFLC than did HF-HD and OL-HDF. MCO-HD and HF-HD showed comparable RRs for β2-microglobulin. No significant difference in the RRs for κFLC and FGF-23. MCO HD showed improved clearances for FLCs when compared to HF-HD or OL-HDF
Cho et al. [43]	2019	Prospective cohort study, HF-HD vs. MCO-HD over a 12-month follow-up	57	Improved reduction rate of FLCs, β2-microglobulin, with no significant changes in long-term effects
Arrascue et al. [82]	2022	Randomized control trial, HDF vs. HDx with 24-week follow up	43	Significant decrease in YKL-40 and reduced use of ESAs in the HDx arm. No significant differences in β2-microglobulin, FGF-23, FLCs and inflammatory marker removal
Cordeiro et. al [83]	2020	Prospective crossover trial; switch from HF-HD to HDF and MCO-HD for 4 weeks	16	β2-microglobulin clearance with on HDF and MCO-HD was higher compared to HF-HD treatment
Belmouaz et al. [84]	2018	Retrospective study, online HDF switched to MCO-HD	10	No significant differences in removal of albumin, urea, creatinine, β2-microglobulin and myoglobin
Lindgren et. al [85]	2020	Prospective controlled single-center cross-over study; MCO-HD vs. online HDF	16	No differences in RR of middle molecules between the two treatments
Lim et al. [52]	2020	Randomized control trial, HF-HD vs. MCO-HD over 12 weeks of treatment	49	Removal of kappa and lambda FLCs was greater for MCO dialyzer than high-flux dialyzer. Higher scores in the domains of physical functioning and physical role in QOL form. Lower scores for morning pruritus distribution and less frequent scratching during sleep
Weiner et al. [46]	2020	Randomized control trial, MCO-HD vs. HF-HD over a 24-week period	172	Higher reduction rate for FLCs, β2-microglobulin, TNF-α, but not for IL-6. Similar predialysis albumin level after 24 weeks of treatment
Krishnasamy et al. [48]	2020	Multicenter, cross-over, longitudinal study, 4-weeks with HF-HD followed by a 24-week period with MCO-HD and then 4 weeks with HF-HD	89	No significant reduction in serum albumin. No improvement in restless leg syndrome, quality of life. Sustained reduction in FLCs in the first 2 weeks after MCO-HD initiation with consequent increase after return to HF-HD
Sevinc et al. [45]	2020	Randomized control trial, HF-HD for 3 months followed by MCO-HD for 3 months or vice-versa	52	Higher reduction rate and post-dialysis levels of β2-microglobulin, FLCs and myoglobin compared to HF-HD. No difference in inflammatory markers. VEGF levels were lower in the MCO group
Belmouaz et. al [5]	2020	Cross-over prospective study, 3 months MCO-HD vs. 3 months HF-HD	40	Higher removal of myoglobin, β2-microglobulin, FLCs, FGF23, homocysteine, with moderate hypoalbuminemia
Yeter et al. [40]	2020	Comparative study, LF-HD vs. HF-HD vs. MCO-HD	42	Reduction in CRP with MCO membranes in a subgroup analysis of 19 patients with high CRP at baseline. No differences in oxidative stress
Reis et al. [6]	2021	Retrospective study, switch to MCO-HD for 2 months, then return to HF-HD	15	Reduction in mean α1-acid glycoprotein and lower median pre-dialysis prolactin concentration during MCO-HD period. β2-Microglobulin increased in the post-MCO phase. Significant reduction in albumin during MCO phase
Perez-Alba et al. [86]	2020	Case series, switched to home HD with MCO	7	β2-microglobulin significantly reduced over 12-month follow-up period, without significant albumin loss and with less use of phosphate binders
Rambabova et al. [87]	2020	12-week observational pilot study, HDx vs. HF-HD	4	Higher average removal rate for β2-microglobulin, myoglobin, FLC-k, and FLC-λ during the 3 months
Cozzolino et. al [29]	2021	Prospective, open label, controlled, cross-over pilot study HDx vs. HD	20	Significant reduction in serum albumin concentration without clinical symptoms of hypoalbuminemia or need for intravenous albumin administration. Reduction of IL-1β and IL-6 levels, while TNF-α levels remained unchanged
Sanabria et. al [54]	2021	Multicenter, observational cohort study, HF-HD switched to HDx for 1 year	81	Decreased hospitalization rate and mean dose of ESAs and iron supplementation after switch to HDx
Catar et al. [88]	2021	Cross-over randomized multicenter comparison, HF-HD vs. MCO-HD	48	MCO treatments modulate proinflammatory mediators and TNF-signaling activation, leading to reduced endothelial maladaptation, VEGF production and angiogenesis
Eiamcharoenying et al. [89]	2022	Randomized controlled trial, MCO-HD vs. mixed dilution online HDF	14	Significant increase in RR of α-1 M and lambda FLC, without significant changes in albumin levels
Blackowicz et al. [90]	2022	Post-hoc analysis of randomized control trial [44], HF-HD vs. MCO-HD	172	Reduced hospitalization rate by 45% in the MCO group with lower average annual estimated costs
Maduell et al. [38]	2022	Prospective study, analysis of 4 MCO membranes vs. HF-HD and HDF	23	All four MCO dialyzers showed similar efficacy in removing myoglobin, κFLC, prolactin, α1-microglobulin and λFLC. RRs with MCO membranes were superior to HD and slightly inferior to HDF treatment. Albumin loss with HF-HD dialyzers was < 1 g and between 1.5 and 2.5 g in HDx and HDF
Krieter et al^50^	2023	Randomized controlled trial in HD patients, comparing Elisio-17HX to Theranova 400 MCO membranes	6	Elisio-17HX achieved slightly lower reduction ratios for β_2_-microglobulin (71.8 ± 6.0 vs. 75.3 ± 5.8%; *p* = 0.001), myoglobin (54.7 ± 8.6 vs. 64.9 ± 8.7%; *p* < 0.001), and kappa-FLC (62.1 ± 8.8 vs. 56.3 ± 7.7%; *p* = 0.021). λFLC reduction ratios were not different between Elisio-17HX and Theranova (28.4 ± 3.9 vs. 38.7 ± 13.4%; *p* = 0.069). The albumin loss of Theranova was considerably higher (2.14 ± 0.45 vs. 0.77 ± 0.25 g; *p* = 0.001)

CRP C-reactive protein; ESA erythropoietin-stimulating agent; FGF23 fibroblast growth factor 23; λFLC lambda free light chain; κFLC kappa free light chain; HDx expanded hemodialysis; HDF hemodiafiltration; HF-HD high flux hemodialysis; IL-6 interleukin 6; LF-HD low flux hemodialysis; MCO medium cut-off; QOL quality of life; OL-HDF online hemodiafiltration; RR reduction rate; TNF tumor necrosis factor; YKL-40 chitinase‐3-like protein 1; VEGF vascular endothelial growth factor

### Potential clinical and socio-economic impact of expanded HD

Accumulation of uremic toxins in ESKD patients is associated with physical symptoms (fatigue, itching, restless leg syndrome) and reduced quality of life; thus efficient removal may improve symptoms and patients’ quality of life.

In a Colombian prospective, multicenter observational study including 992 patients, Alcaron et al. showed that 3 of 5 domains of the Kidney Disease Quality of Life 36-Item Short Form Survey (KDQoL-SF36) improved after the switch from high flux-HD to expanded hemodialysis for 12 months [51]. Moreover, the prevalence of patients with restless leg syndrome was significantly reduced at 12 months (22.1 vs. 10%, *p* < 0.001) [51]. Similar results were shown by Lim et al. who enrolled 49 patients on chronic HD randomized to either a medium cut-off membrane or high flux-HD [52]: patients in the medium cut-off group reported higher scores in the physical functioning and physical role domains of KDQoL-SF36, while they reported reduced scores for morning pruritus and lower frequency of scratching during sleep [52].

Further studies have focused on the effect on inflammation and oxidative stress markers that is associated with endothelial dysfunction, vascular calcification, increased cardiovascular risk, malnutrition and mortality [14]. Zickler et al. reported reduced expression of pro-inflammatory TNF-α and IL-6 mRNA in peripheral leukocytes in patients treated with medium cut-off membranes compared to high flux-HD, even though cytokine levels over the 12 weeks of follow-up were not significantly different [41]. Studies on the role of oxidative stress gave controversial results [5, 29, 45]. A recent clinical study by Lee and colleagues did not show any significant differences on several cardiovascular parameters such as echocardiography, changes in brachial-ankle pulse wave velocity between expanded HD and online HDF [53]. Moreover, the coronary artery calcium score over 1 year increased in the expanded HD group [53].

A relevant point is also that expanded HD has been associated with cost reduction. In fact, extended HD does not require a large volume of fluids as compared to HDF, and if lower hospitalization and hospital stay rates per patient-year, found in a cohort of 81 patients are confirmed this could lead to considerable savings [54]. Moreover, less use of erythtopoietin-stimulating agents and iron supplementation has been reported, suggesting that the improved removal of inflammatory mediators may improve iron metabolism and erythtopoietin-stimulating agent resistance.

### Middle cut-off membranes in the context of AKI

Acute kidney injury is a complex clinical syndrome associated with high costs of hospitalization, mortality, and long-term complications (progression to CKD, cardiovascular diseases) [17, 19]. Sepsis is one of the main causes of AKI [55, 56]. Mortality in septic patients with AKI requiring renal replacement therapy is still very high, reaching 60% [55]. One of the hallmarks of sepsis is the imbalance between pro- and anti-inflammatory cytokines, leading to multi-organ dysfunction [57]. For this reason, critically ill patients with sepsis-associated AKI may benefit from extracorporeal blood purification therapies in an effort to remove inflammatory mediators and decrease cytokine gradient between blood and tissues, leading to restoration of immune balance and reducing the risk of organ failure [58]. Among the proposed modalities, high-volume hemofiltration, high cut-off membranes and adsorption cartridges have been proposed, with controversial results. High cut-off membranes with a cutoff up to 60 kDa demonstrated higher cytokine removal compared to conventional HD membranes [59–62]. However, the albumin and antibiotic loss associated with their use, in addition to the limited effects on hemodynamic support and overall mortality, have limited their application in clinical practice [63].

Recently, the EMiC2 filter (Fresenius, Bad Homburg, Germany) has been introduced in clinical practice: it is a polysulfone-based membrane with a cutoff of 45 kDa. These characteristics make it suitable for the removal of middle molecules, such as k-free light chains (23 kDa), myoglobin (17 kDa) and β-2microglobulin (17 kDa) [64–66] and some studies reported higher removal of IL-6 and IL-8 compared to standard high flux membranes [67, 68]. Lumlergul and colleagues performed a prospective observational study and analyzed the actual clearance of middle molecular weight molecules (IL-1β, IL-1, IL-2, IL-4, IL-6, IL-8, IL-10, TNF-α, VEGF, EGF, MCP-1) in vivo, using the EMiC2 filter in 12 critically ill patients with sepsis-associated AKI requiring renal replacement therapy (continuous veno-venous hemodialysis) [69]. The authors showed a significant reduction in plasma concentrations of all analyzed molecules over a 48-h period after continuous veno-venous hemodialysis initiation, except for EGF. However, the effluent clearance rates were low for most cytokines with minimal adsorption effects made by the filter, suggesting that the overall reduced plasma concentration is only partially related to dialytic clearance and that other mechanisms may contribute to the observed changes [69]. Recently, Comoglu et al. analyzed the effects of medium cut-off membranes in a cohort of 38 patients with sepsis-associated AKI who received 2 hemodialytic treatments with 2 different membranes (19 patients started treatment with a high flux membrane and then switched to a medium cut-off membrane, the remaining 19 patients were first treated with a medium cut-off membrane, and then switched to a high flux membrane) [70]. The reduction of TNF-α, IL-6 and IL-1β was significantly higher during HD treatment with medium cut-off membranes than with high flux, while no differences were reported among markers of oxidative stress [70]. Recently, a single center crossover randomized study enrolling 20 patients with septic shock and AKI stage 3 comparing the EmiC2 filter with a high-flux membrane showed significant hemodynamic improvement and greater β2 microglobulin removal in the EmiC2 group [71].

Moreover, blunting the hyperinflammatory state characterized by the so-called “cytokine storm” has been one of the main targets in treating severe forms of COVID-19. In this setting, the use of HDF or expanded HD was proposed to maximize cytokine clearance in HD patients [72, 73]. Serrano Salazar et al. performed a prospective observational study including 18 patients with COVID-19 infection who required hemodialysis and compared online HDF, expanded HD and a control group of 8 HD patients without COVID-19 infection with regard to removal of several cytokines, β2 microglobulin and albumin [74]. Expanded hemodialysis provided the best clearance for TNF-α (67 vs. 54% in online HDF) and β2 microglobulin during HD sessions, without significant albumin loss [74]. In addition, mortality was higher in the HDF group (57.1 vs. 18.2%), albeit not statistically significant, due to the limited sample size [74].

A further indication may be kidney injury in multiple myeloma related to cast nephropathy in which free light chain removal is a therapeutic target. In this setting, few case reports showed that medium cut-off membranes were effective in the removal of free light chains favoring kidney function recovery [75].

Finally, myoglobin removal may be pivotal in patients with AKI secondary to rhabdomyolysis, a common complication during crush syndrome and other conditions (trauma, ischemia, hyperthermia, drug intoxication). The use of medium cut-off membranes in such conditions has been reported in both intermittent and continuous dialysis modalities [76, 77]. Jerman et al. reported the efficacy of high cut-off and medium cut-off membranes in removing myoglobin in 15 patients with AKI and rhabdomyolysis when comparing these filters to specific cytokine adsorbers [77].

Taken together, the present evidence on the use of medium cut-off membranes in AKI is limited and mainly consists of observational studies focusing on the clearance of middle molecules, most of them not powered to assess association with clinical outcomes. In addition, AKI patients are extremely heterogeneous; further work is required to better assess the role of such membranes in clinical practice.

## Conclusions

The recent development of medium cut-off membranes characterized by high-retention-onset and the introduction of the concept of expanded HD represents a step forward in personalized approaches in HD. Medium cut-off membranes are able to remove medium–high uremic toxins, such as β2-microglobulin, free light chains, myoglobin and others, that are associated with inflammation, cardiovascular events and mortality in both acute and chronic dialysis patients. Since the current evidence is limited, further larger and long-term studies are needed.
